# Assessing the Gaps and Needs of Healthcare Professionals Caring for Persons Living with Behavioral and Psychological Symptoms of Dementia

**DOI:** 10.1002/alz70860_102064

**Published:** 2025-12-23

**Authors:** Sylvia Peng, Tamara J. LeCaire, Molly Schroeder, Tammi Albrecht, Jennifer Landeta‐Vidal, Uriel Paniagua, Cynthia M. Carlsson, Art Walaszek

**Affiliations:** ^1^ UW Madison School of Medicine and Public Health, Madison, WI, USA; ^2^ Wisconsin Alzheimer's Institute, Madison, WI, USA; ^3^ Wisconsin Alzheimer's Institute, University of Wisconsin School of Medicine and Public Health, Madison, WI, USA; ^4^ Wisconsin Alzheimer's Institute, University of Wisconsin School of Medicine and Public Health, Madison, WI, USA

## Abstract

**Background:**

In 2023, there were 6.7 million Americans living with Alzheimer's disease. The majority of persons living with dementia (PLWD) receive dementia care from primary care providers; however, primary care providers have reported limited training related to behavioral and psychological symptoms of dementia (BPSD) and lacking self‐efficacy to manage BPSD. The majority of persons living with dementia experience at least one BPSD, and these symptoms can worsen quality of life and decrease survival. The purpose of our study was to assess the gaps and needs that healthcare professionals face in the identification and management of BPSD, with the goal of informing and tailoring educational programs to address those gaps.

**Method:**

A survey was content piloted and then disseminated to healthcare providers who are part of the Wisconsin Alzheimer's Institute (WAI) Dementia Diagnostic Clinic Network. Additional respondents were identified through snowball sampling, wherein initial respondents were asked to refer other persons to be surveyed. Respondents were asked to describe their practices with respect to BPSD, to identify gaps and needs for identifying and managing BPSD, and to rate their knowledge of dementia and their skills caring for people living with BPSD. Quantitative and qualitative data will be summarized using mixed methods approaches.

**Result:**

The survey was completed by 45 healthcare professionals. Many respondents indicated gaps in their individual (53%) and community's (56%) ability to identify and manage BPSD. Gap areas identified included needing an accurate diagnosis, knowledge base related to managing BPSD, lack of access to consultation/resources, and staff training and turnover at facilities. For example, 31% of respondents identified lack of confidence managing sexually inappropriate behaviors, a potential target for future training. These results are preliminary and data collection is ongoing.

**Conclusion:**

Gaps in the ability to identify and manage BPSD were prevalent among healthcare professionals surveyed. Gaps suggest that healthcare professionals could benefit from educational programming on BPSD, tailored to their needs. The goal of educational programming should be to increase healthcare professionals’ knowledge and confidence in identifying and managing BPSD so that they can help improve the lives of PLWD.

